# Predictive value of suvmax changes between two sequential post-therapeutic FDG-pet in head and neck squamous cell carcinomas

**DOI:** 10.1038/s41598-020-73914-3

**Published:** 2020-10-07

**Authors:** Thomas M. Stadler, Martin W. Hüllner, Martina A. Broglie, Grégoire B. Morand

**Affiliations:** 1 Department of Otorhinolaryngology - Head and Neck Surgery, University Hospital Zurich, Frauenklinikstrasse 24, 8091 Zurich, Switzerland; 2 University of Zurich, Zurich, Switzerland; 3 Department of Nuclear Medicine, University Hospital Zurich, Zurich, Switzerland

**Keywords:** Cancer, Surgical oncology, Oncology

## Abstract

18-flurodesoxyglucose position emission tomography (FDG-PET) with computed tomography (CT) or magnetic resonance imaging (MRI) is a broadly accepted tool for pretherapeutic staging and post-therapeutic assessment of response. The prognostic value of sequential post-therapeutic FDG-PETs and the impact of change in metabolic activity has been scarcely reported so far. We hypothesized that an increase in metabolic activity (as measured by maximum standardized uptake value, SUVmax) would be predictive for recurrence. We retrospectively assessed all oral, oropharyngeal, laryngeal, and hypopharyngeal squamous cell carcinoma patients treated at the Department of Otorhinolaryngology—Head and Neck Surgery, University Hospital Zurich between April 1st, 2010 and September 30th, 2018 (N = 337). After a negative post-treatment FDG-PET at 3 months, we measured the SUVmax of the local tumor area and the regional lymph nodes on follow-up FDG-PET at 9 months. We then correlated SUVmax difference between 9 and 3 months with tumor recurrence using Kaplan Meier analysis. During follow-up, 68 patients (20.2%) had local recurrence and 53 had regional recurrence (15.7%) at a median time of 9.0 (IQR 4.25–14) and 7.0 (IQR 5.25–23) months, respectively. An increase in local and/or regional SUVmax from the 3 months to the 9 months post-therapeutic FDG-PET resulted in a poorer recurrence-free survival (Log rank, *P* = 0.001, for both). An increase in local SUVmax between 3 and 9 months was associated with a hazard ratio of 4.17 for recurrence (95%CI 1.89–9.2, *P* = 0.0003). In conclusion, an increase in metabolic activity/SUVmax between two post-therapeutic FDG-PETs requires a histological examination as it is associated with tumor recurrence.

## Introduction

18-fluorodeoxyglucose positron emission tomography (FDG-PET) with computed tomography (CT) or magnetic resonance (MR) imaging has become a broadly accepted imaging tool in routine clinical oncology. Adding FDG-PET to the staging process results in improved nodal classification, detection of distant metastases, and contralateral nodal disease^1–3^. FDG-PET is also a valuable asset for primary tumor identification in patients with carcinoma of unknown primary (CUP) of the head and neck^4^. FDG-PET can also be used as response assessment in the post-therapeutic setting, as a negative FDG-PET after chemoradiation can exclude with a high negative predictive value (NPV) residual locoregional disease^5,6^. Quantitative assessment of metabolic activity by e.g. maximum standardized uptake value (SUV_max_) is in turn useful for identifying tumors with a more aggressive phenotype and hence patients that may require more intensive treatment protocols^7–10^.

In the post-treatment surveillance setting, there are no internationally defined follow-up imaging strategies and they differ widely between institutions and countries^11,12^. The normal anatomy of the head and neck becomes distorted by surgery and/or chemoradiation. Distinguishing residual or recurrent tumor from treatment related changes is often virtually impossible on anatomical imaging studies such as CT and MRI^13^. At our institution, it has become our internal policy to perform posttherapeutic FDG-PETs at three months and at nine months after definitive treatment for patients with advanced stage disease.

While the value of a single FDG-PET in the pretherapeutic and posttherapeutic setting has been described, the prognostic value of changes in metabolic activity on sequential posttherapeutic FDG-PETs has been scarcely reported so far. We hypothesized that, after a negative posttherapeutic FDG-PET at three months, an increase in metabolic activity during follow-up was associated with tumor recurrence, whereas the metabolic activity of post-therapeutic inflammatory changes would decrease over time.

## Materials and methods

### Study population

**Figure 1:**
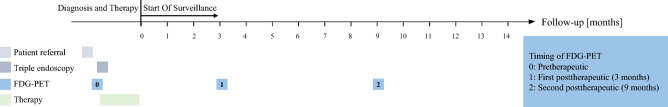
Graphical representation of pre- and post-treatment algorithm at our clinic. Upon referral, patients received triple endoscopy. FDG-PET is obtained as indicated. Patient are treated according to our multidisciplinary tumor board decision. After definitive therapy, patients enter post-therapeutic follow-up for 60 months. If FDG-PET was performed before therapy, this is repeated in the post-therapeutic setting at 3 and 9 months.

After local ethics review board approval by the *Kantonale Ethikkommission Zürich*, we retrospectively assessed all patients with squamous cell carcinoma of the oral cavity, oropharynx, larynx, and hypopharynx treated at the Department of Otorhinolaryngology – Head and Neck Surgery of the Zurich University Hospital, Zurich, Switzerland between April 1st 2010 and September 30th, 2018. Study methods were carried out in accordance with the relevant guidelines and regulations. Informed consent was obtained from all subjects. Inclusion criteria were available pre-therapeutic FDG-PET/CT or FDG-PET/MR images as well as post-therapeutic FDG-PET/CT or FDG-PET/MR. According to our institutional policy, pretherapeutic FDG-PET was obtained for all patients with advanced stage disease (stage III / IV, ≥ T3, ≥ N2a/b). However, we also included a few patients with < T3 or < N2a referred externally with an already available FDG-PET. After completion of treatment, posttherapeutic FDG-PETs were performed at three and nine months of follow-up (Fig. 1). The study cohort was then divided into four groups according to tumor site, analyzing oral, oropharyngeal, laryngeal, and hypopharyngeal cancer separately. Patients without further imaging after initial diagnosis were excluded. Only patients treated in curative intent were included.

All patients were staged according to the *Union Internationale Contre le Cancer (UICC)*, TNM staging for head and neck cancer, 7th edition, 2010^14^. All cases were discussed at the local interdisciplinary tumor board and treated according to the NCCN Guidelines^15^.

Detailed data on age, gender, tumor subsite, and risk factors including smoking, drinking habits, and human papilloma virus (HPV) status were obtained. In oropharyngeal cancer, immunohistochemical expression of p16 was assessed and in positive cases polymerase chain reaction (PCR) for HPV was used to evaluate HPV status of tumor biopsy samples^16^. Tobacco use was defined as a current daily consumption or history of daily consumption of cigars or cigarettes. Alcohol abuse was defined as a daily intake of more than 20 g of ethanol at least five days a week^17^.

### FDG-PET/CT or -/MR acquisition

Detailed FDG-PET acquisition protocol have been reported previously^18^. After a fasting time of at least four hours, patients were injected with a standardized dose of 3.5 MBq of FDG per kilogram body weight (PET/CT) or 3.0 MBq per kg body weight (PET/MR), and from 2017 on with a body mass index-adapted, body weight-dependent dosage protocol for PET/MR^19^. All doses were prepared an injected with an automatic PET infusion system (Medrad Intego, Bayer Healthcare), the prescribed dose was met by ± 5% in all subjects. Glucose level was measured and ranged 4–12 mmol/l before imaging.

Resting time after injection was one hour. During this period, patients were advised to remain in lying position without talking in order to minimize muscular FDG uptake. All subjects met the uptake time of 60 min + /- 5 min. Patients were kept warm prior to tracer injection and throughout the uptake period to diminish FDG accumulation in brown adipose tissue. Most patients received either iodinated or gadolinium-based contrast medium. An integrated Discovery VCT PET/CT system (GE Healthcare, Waukesha, WI, USA), a Discovery PET/CT 690 (GE Healthcare), or a hybrid PET/MRI system (Signa PET/MR, GE Healthcare) was used for image acquisition. All PET images were reconstructed with the same algorithm (ordered subset expectation maximization).

For attenuation correction in PET/CT, transmission data from a standard low-dose CT scan was used. For attenuation correction in PET/MR, a two-point Dixon-type three-dimensional (3D) gradient dual-echo MR pulse sequence (Liver acquisition with volume acceleration, LAVA-flex) was used^20^. All images used for attenuation correction were without i.v. contrast material.

The regionalized anatomical CT and MR imaging protocol for the head and neck used standard acquisition parameters for head and neck imaging, including adapted field of view (e.g., MR field of view range: 24 to 50 cm, depending on respective pulse sequence) and increased matrix size (e.g., MR matrix size range: 256 × 192 to 320 × 256 pixels, depending on respective pulse sequence), as published previously^20^.

CT emission data were iteratively reconstructed (matrix size 256 × 256 pixels, 3D TOF ordered subset expectation maximization (OSEM) with 3 iterations and 18 subsets, with point spread function, 4.7 mm full width at half maximum, 1:4:1 weighted axial filtering.

The SUV_max_ of the primary tumor was obtained under supervision of a dually board-certified nuclear medicine physician/radiologist (MWH). Moreover, in patients with clinically positive nodal status, the SUV_max_ of the dominant (that is with the highest SUV_max_) lymph node was recorded as well. SUV_max_ was calculated automatically using a standard formula [maximum activity in region of interest ÷ (injected dose × body weight)]. Correct analysis of FDG uptake was ensured through side-by-side reading of the corresponding CT or MR images of the tumor in the axial, coronal, and sagittal plane. Borders of regions of interest (ROI) were set by manual adjustment to exclude adjacent physiologic FDG-avid structures.

### Primary analysis (Fig. 2)

**Figure 2:**
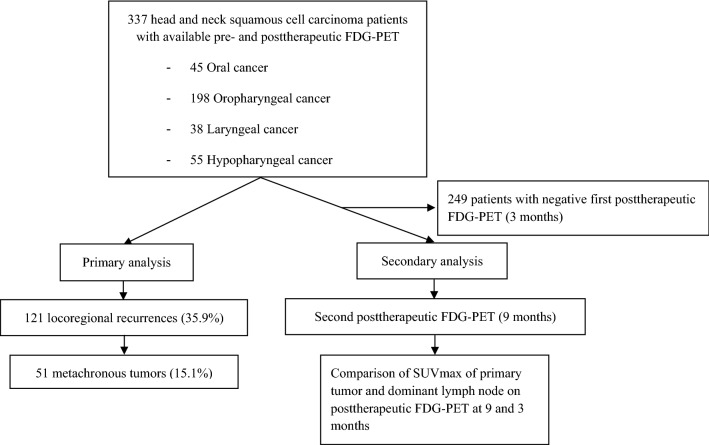
Flow chart showing how patients were included in the primary and secondary analysis.

For the primary outcome analysis, we included all patients in the analysis. Local recurrence was defined as presence of tumor confirmed by histopathology in the primary tumor site after completion of curatively intended treatment. Regional recurrence was defined by cytologically and/or histologically confirmed presence of malignant disease in the cervical lymph nodes after completion of curatively intended treatment. Time to local and regional recurrence was calculated in months from the completion of curative therapy (time zero) unto the date of histological or cytological proof of local or regional recurrence.

All metachronous tumors had to be confirmed by histopathology and had a location different from the primary tumor for which the patients were treated. Time to metachronous tumor was calculated in months from the completion of curative therapy (time zero) unto the date of histological or cytological proof of second primary.

### Secondary analysis (Fig. 2)

#### Analysis of sequential posttherapeutic FDG-PET

For the secondary outcome analysis, we aimed to evaluate the prognostic value of the second posttherapeutic FDG-PET done at 9 months after completion of treatment. Therefore, we only included patients showing a recurrence detected by the second posttherapeutic FDG-PET at 9 months. Median time difference between the end of treatment and first posttherapeutic FDG-PET at 3 months was 92.5 days (IQR 83 – 111.5). Median time difference between end of treatment and second posttherapeutic FDG-PET after nine months was 261.5 days (IQR 239.75 – 277). The median time difference between the second FDG-PET at nine months and pathological confirmation of tumor recurrence was 41 days (IQR 22.5 – 86).

Therefore, to accommodate for slight variations when calculating the time to recurrence and owing to slight variations in the timing of three months and nine months FDG-PET, we included all recurrences recorded to occur between 8 and 14 months after completion of treatment (time zero). All recurrences occurring before and after this time frame were excluded from the secondary analysis.

All patients included in the secondary analysis therefore had showed a complete metabolic response (score of two or less according to the Hopkins criteria)^21^ on the first posttherapeutic FDG-PET at three months after completion of treatment. These patients were then followed-up clinically, another FDG-PET was acquired at nine months. We then assessed the SUV_max_ of the local tumor area and dominant regional lymph node on the FDG-PET at nine months after completion of treatment. The SUV_max_ at three months was subtracted from the SUV_max_ at nine months. The difference (> 0 – increase vs. ≤ 0 – decrease-stable) was then correlated with recurrences (local and regional, respectively) occurring or detected after the second post-therapeutic FDG-PET at nine months.

### Statistical analysis

For continuous variables, distribution was evaluated for normality according to Gauss’ theorem^22^. For non-normally distributed variables, median and interquartile range (IQR) are given. For normally distributed variables, mean and standard deviation (SD). To compare the distribution among samples, the one-way ANOVA and the non-parametric Kruskal–Wallis test were used for continuous variables, according to their distribution (normal vs. non-normal, respectively). For nominal variables, the chi-square test was used. Main outcome measures of the study were calculated using a Cox regression model. Results are expressed in hazard ratio (HR) with the 95% confidence interval provided (95% CI). Survival curves were built according to the Kaplan–Meier method. The log-rank test was performed to compare survival among factors. A P-value lower than 0.05 was considered to indicate statistical significance. Statistical analyses were performed using SPSS 25.0.0.0 software (IBM, Armonk, NY, USA).

## Results

### Patient and tumor characteristics

A total of 337 consecutive patient were included. The mean age at diagnosis was 66 (SD 11.2). Patients were predominantly male (248/337, 73.6%). Forty-six (13.6%) had oral cavity cancer, while 198 (58.8%), 38 (11.3%), and 55 (16.3%) had oropharyngeal, laryngeal, and hypopharyngeal cancer, respectively. Overall, 264 out of 337 (78.8%) of the patients had advanced disease; 179 patients (53.1%) had cT3-cT4 tumors in comparison to 158 (46.9%) of patients with cT1-cT2 tumors. Clinical nodal status was positive in 232 patients (68.8%), of which 91 were staged cN1 (27.0%), 76 cN2a-b (22.5%) and 65 cN2c-cN3 (19.2%). Median follow-up time for all patients was 22.0 months (IQR 10 – 40). Most patients received primary chemoradiation (67.9%, 229/337), whereas 108 patients were treated surgically with (55.5%, 60/108) or without (44.5%, 48/108) adjuvant radiation (32.1%, 108/337) (Table 1).

**Table 1:** Patient demographics and clinical characteristics.

Variable		All patients N = 337	Oral N = 46	Oropharynx N = 198	Larynx N = 38	Hypopharynx N = 55	*P *value (Oral vs. Oropharynx vs. Larynx vs. Hypopharynx)
**Age**							
Years	Mean (SD)	66 (11.2)	60 (15.6)	69 (10.1)	63 (8.9)	64 (9.6)	0.001
**Gender**							
Male	n (%)	248 (73.6%)	30 (65.2%)	139 (70.2%)	31 (81.6%)	48 (87.3%)	0.025
Female	n (%)	89 (26.4%)	16 (34.8%)	59 (29.8%)	7 (18.4%)	7 (12.7%)	
**Smoking**							
	Yes (%)	252 (74.8%)	28 (60.9%)	102 (51.5%)	37 (97.4%)	53 (96.4%)	< 0.001
	No (%)	85 (25.2%)	18 (39.1%)	96 (48.5%)	1 (2.6%)	2 (3.6%)	
Pack years	Median (IQR)	45 (30 – 60)	46.5 (30 – 60)	45 (30 – 60)	54 (40 – 80)	40 (25–62.5)	< 0.001
Alcohol abuse	Yes (%)	137 (40.7%)	25 (54.3%)	130 (65.7%)	19 (50.0%)	26 (47.3%)	0.038
	No (%)	200 (59.3%)	21 (45.7%)	68 (34.3%)	19 (50.0%)	29 (52.7%)	
p16 IHC	Positive			96 (48.4%)			n/a
	Negative			93 (46.9%)			
	n/a			9 (4.5%)			
**T-classification**							
cT1 – cT2	n (%)	158 (46.9%)	27 (58.7%)	93 (46.9%)	15 (39.5%)	23 (41.8%)	0.004
cT3 – cT4	n (%)	179 (53.1%)	19 (41.3%)	105 (53.1%)	23 (60.5%)	32 (58.2%)	
**N-classification**							
N0	n (%)	105 (31.2%)	24 (52.2%)	51 (25.7%)	20 (52.6%)	10 (18.2%)	< 0.001
N1	n (%)	91 (27.0%)	7 (15.2%)	73 (36.9%)	4 (10.5%)	7 (12.8%)	
N2a – N2b	n (%)	76 (22.5%)	10 (21.7%)	42 (21.2%)	5 (13.2%)	19 (34.5%)	
N2c – N3	n (%)	65 (19.3%)	5 (10.9%)	32 (16.2%)	9 (23.7%)	19 (34.5%)	
**Therapy**							
Surgery	n (%)	48 (14.3%)	18 (39.1%)	29 (14.6%)	0 (0.0%)	1 (1.8%)	< 0.001
Surgery + RT	n (%)	60 (17.8%)	27 (58.7%)	30 (15.2%)	0 (0.0%)	3 (5.5%)	
Radiotherapy	n (%)	229 (67.9%)	1 (2.2%)	139 (70.2%)	38 (100%)	51 (92.7%)	
**Recurrence**							
Local	n (%)	68 (20.1%)	12 (26.1%)	15 (7.5%)	12 (31.5%)	29 (52.7%)	0.001
Regional	n (%)	53 (15.7%)	15 (32.6%)	20 (10.1%)	5 (13.1%)	13 (23.6%)	0.032
**Time to recurrence (months)**							
Local	Median (IQR)	9 (4.2 – 14)	6.5 (4 – 25)	9 (6 – 12)	9 (5.5 – 14.7)	12 (4.5 – 14.5)	0.810
Regional	Median (IQR)	7 (5.2 – 23)	7 (4 – 7)	6 (5 – 12)	17 (6 – 26)	14 (6.5 – 22.5)	0.105
*Follow up*	Median (IQR)	22 (10 – 40)	32.5 (16.7 – 52.2)	16 (7 – 27.7)	31 (22.2 – 62.5)	29 (20 – 57)	0.001

SD: standard deviation; IQR: interquartile range; IHC: immunohistochemistry; n/a: not available; RT: Radiotherapy.

ANOVA normally distributed variables. Kruskal–Wallis test for non-normally distributed continuous variables. Chi-square test for nominal variable. Log-rank test for time-dependent variable (local and regional recurrence).

### Primary analysis

#### Pattern and timing of recurrences

In the whole study cohort (n = 337), there were 121 locoregional recurrences (35.9%), of whom 68 (20.2%) had local recurrence and 53 (15.7%) had a regional recurrence. The median time to local recurrence was 9.0 months (IQR 4.25 – 14). The median time to regional recurrence was 7.0 months (IQR 5.25–23) (Supplemental Figs. 1 and 2). The vast majority of local and regional recurrences occurred in the first 24 months after completion of treatment (Table 2).

**Table 2:** Time of recurrence.

	Local recurrence	Regional recurrence
Within 12 months	41/68 (60.3%)	32/53 (60.4%)
Within 24 months	60/68 (88.2%)	41/53 (77.4%)
Within 36 months	65/68 (95.6%)	49/53 (92.5%)
Within 48 months	66/68 (97.1%)	50/53 (94.3%)
Within 60 months	68/68 (100%)	53/53 (100%)

**Figure 3:**
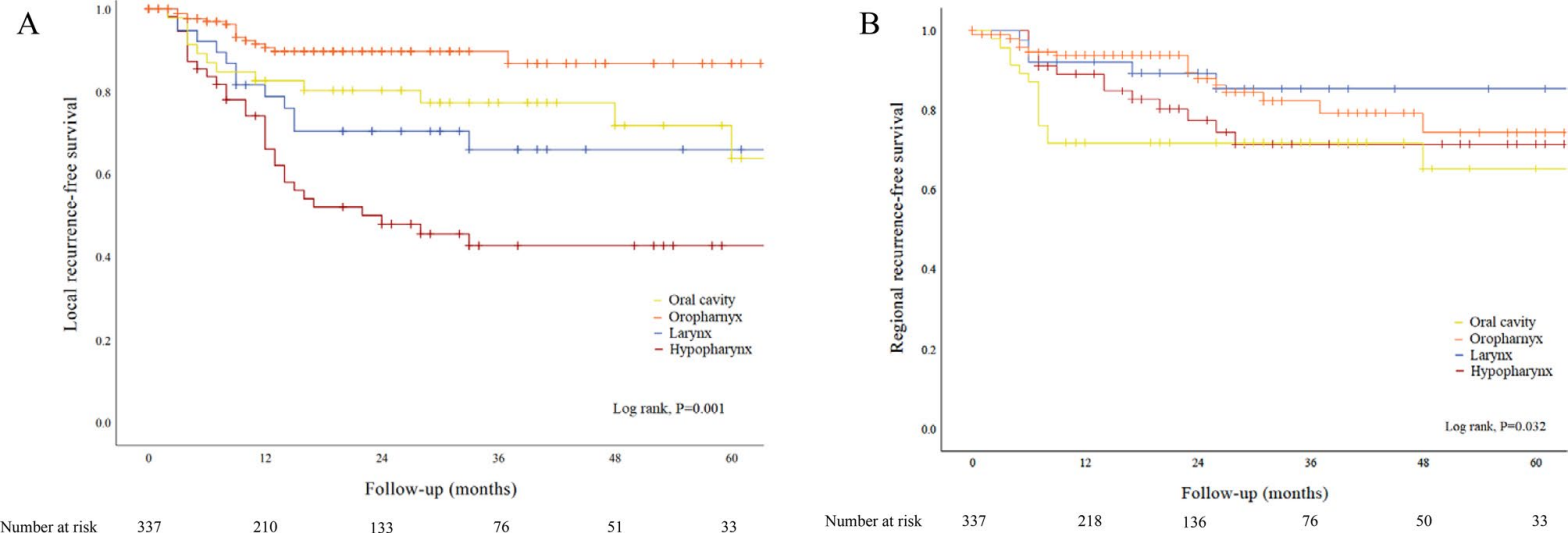
Kaplan–Meier analysis showing local recurrence-free (**A**) and regional recurrence-free survival (**B**) in patients according to primary tumor site, respectively. Both survival rates were different in a statistically highly significant manner (Log rank, *P* = 0.001 and *P* = 0.032, respectively).

When comparing local and regional recurrence by site, there was a highly significant difference in tumor recurrence (Log rank test, *P* = 0.001 and *P* = 0.032, respectively). Oropharyngeal cancer had the lowest local recurrence rate, meanwhile hypopharyngeal had the highest local recurrence rate, while laryngeal carcinoma had the lowest regional recurrence rate. (Fig. 3, Panel A and B).

#### Rate of metachronous primary tumors

We then assessed the rate of second metachronous primaries and plotted them against time (Supplemental Fig. 3). There were 51 cases of metachronous tumors (15.1%), 45 in smokers (13.3%), and six in non-smokers (1.8%). This difference was statistically significant (HR 2.86, 95%CI 1.17–6.9, *P* = 0.015). Smokers had therefore can approximately three times higher likelihood to develop metachronous primary tumors.

### Secondary analysis

#### Local SUV_max_ temporal changes

**Figure 4:**
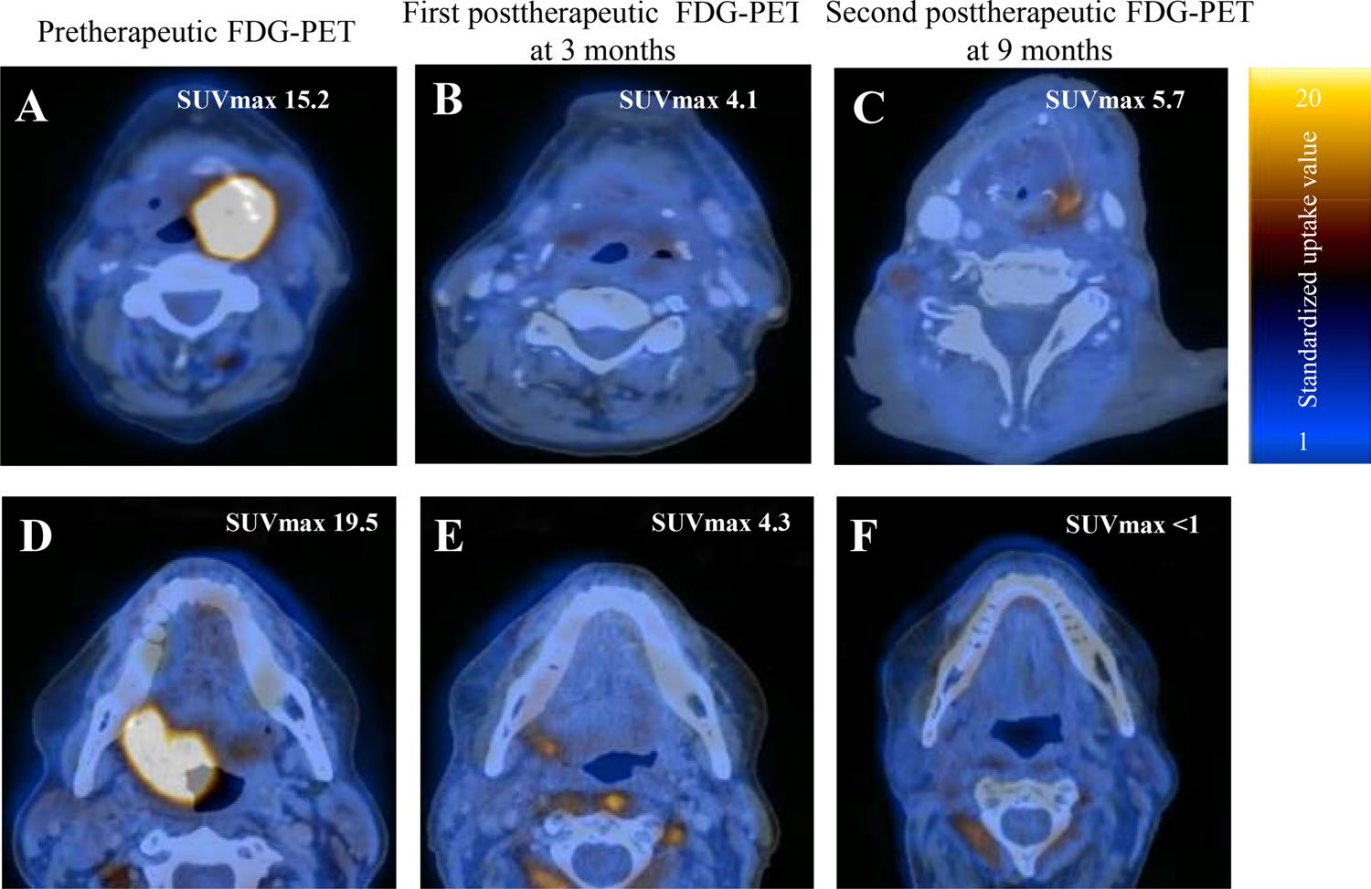
Representative axial fused FDG-PET/CT images demonstrating SUV_max_ changes in patients with head and neck carcinoma. The first patient had hypopharyngeal carcinoma and received chemoradiation. After initial decrease of SUV_max_ between the pretherapeutic scan (**A**) and the first post-therapeutic FDG-PET (3 months) (**B**), there was an increase in SUV_max_ in the second post-therapeutic FDG-PET (9 months) (**C**). Local recurrence was confirmed by histopathology following the 9 months FDG-PET. Other example of oropharyngeal cancer patient. SUV_max_ decreased between the pretherapeutic (**G**), first FDG-PET after 3 months (**H**) to a similar level as shown in the first patient (Panel A and B). In the second post-therapeutic FDG-PET, however, SUV_max_ of primary tumor area further decreased. Patient is free of disease upon last follow-up.

We then evaluated the predictive value of two sequential posttherapeutic FDG-PETs. Figure 4 exemplifies two patients with complete metabolic response at three months (Hopkins < 2). In the first patient (Fig. 4, Panel A-C), the SUV_max_ at nine months slightly increased (from 4.1 to 5.7, 39.0% increase). Histopathological analysis revealed local recurrence. In the second patient (Fig. 4, Panel D-F), SUV_max_ decreased between three and nine months FDG-PET (from 4.0 to < 1 100% decrease). This patient remained free of disease.

**Figure 5:**
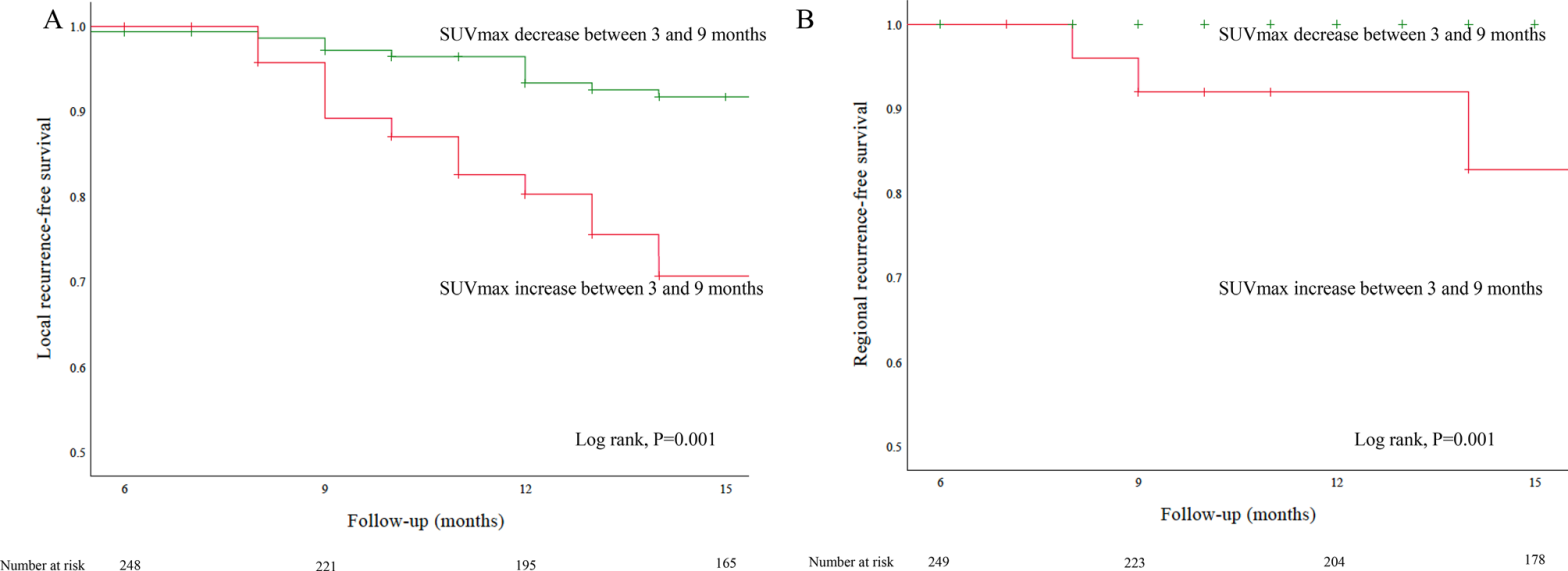
Kaplan–Meier analysis showing local recurrence-free (**A**) and regional recurrence-free survival (**B**) in patients according to tumor site. Both relative recurrence-free survivals were different in a statistically highly significant manner (Log rank, *P* = 0.001, for both).

Overall, there were 22 local recurrences recorded in the time frame for the secondary analysis (see methods). When assessing the local SUV_max_ changes between three and nine months, statistical analysis revealed that an increase in SUV_max_ of the primary tumor area between the second and first post-therapeutic FDG-PET predicted a higher risk of local recurrence (Fig. 5, Panel A, Log rank test, P < 0.001). For disease-specific survival, the difference in survival was not significant if local SUVmax increased or decreased between 9 and 3 months FDG-PET (Log rank test, *P* = 0.155).

Using Cox-regression analysis, we calculated that an increase in local SUV_max_ between three and nine months results in a higher chance for recurrence with a HR 4.17 (95%CI 1.89–9.2, *P* = 0.0003).

Of the patients with local recurrence detected following the second post-therapeutic FDG-PET, 12/22 patients (54.5%) received salvage surgery of their local recurrence. Three (30.0%) of the ten patients without salvage surgery died shortly after the diagnosis of recurrence. One patient had tumor bleeding, one pneumonia and the third died at home without further information. Three patients (30.0%) had bilateral lung metastases. Four (40.0%) had tumors that were inoperable.

Four patients had local and regional recurrence and all these patients underwent successful salvage surgery.

#### Regional SUV_max_ temporal changes

We further evaluated regional recurrences occurring after the second posttherapeutic FDG-PET and examined the changes of metabolic activity of the dominant node between the second and the first posttherapeutic FDG-PET, at nine and three months, respectively. Overall, there were 14 regional recurrences recorded in the time frame for the secondary analysis (see methods).

An increase in SUV_max_ of nodal disease between the second and first post-therapeutic FDG-PET predicted a higher risk of regional recurrence (Log rank test, P < 0.001) (Fig. 5, Panel B).

For disease-specific survival, the difference in survival was not significant if regional SUVmax increased or decreased between 9 and 3 months FDG-PET (Log rank test, *P* = 0.308).

Of the 14 patients who suffered regional recurrence in the defined interval after the second post-therapeutic FDG-PET, six (42.8%) were treated with salvage surgery. The remaining eight (57.2%) patients received chemotherapy due to simultaneous distant metastases. All eight patients had bilateral lung metastases, three had additional bone metastases and one brain metastases as well.

## Discussion

In this retrospective analysis of patients with head and neck cancer, we show that, after a negative first posttherapeutic FDG-PET, an increase in SUV_max_ of the primary tumor and/or lymph node is associated with an increased risk of recurrence. Second, we show that the vast majority of local and regional recurrences occur within two years after completion of treatment, while the occurrence of metachronous second primaries does not fade over time if exposure to risk factors is present.

Our locoregional recurrence rates of about one third of patients is within the range reported in the literature. These vary between 20–50% depending on primary tumor location and stage^23–25^. We included only patients with available pretherapeutic FDG-PET, i.e., with advanced stage, according to our internal policy. The median time to local and regional recurrence was 9.0 and 7.0 months, respectively. Based on this timing and based on the rate of recurrences, we argue that a close monitoring in the first two years is of paramount importance.

Hypopharyngeal carcinoma had the highest local recurrence rate followed by laryngeal carcinoma. For regional recurrence, hypopharyngeal and oral cavity carcinoma had the highest rate, followed by laryngeal carcinoma. However, it should be mentioned that hypopharyngeal carcinoma was the site with the most advanced disease at presentation. Interestingly, patients with oral cancer had a less advanced disease stage at diagnosis, but had a high rate of regional recurrence. Again, the rate of local and regional recurrences in the literature varies widely among different primary tumor locations and initial stage, with the worst outcome typically seen in hypopharyngeal carcinoma, as in our study^26^.

The risk of developing a metachronous second carcinoma turned out to be almost constant over the time after treatment of the first tumor. In our cohort, 47/337 patients (13.9%) developed a metachronous second primary carcinoma within 60 months. In the literature this rate varies between 7 – 15% after five years^27–29^.

The risk of a second primary was much higher in smokers, indicating that this population might benefit from prolonged follow-up. Whether this strategy leads to significant survival improvement remains hypothetical. Further, our data does not allow us to recommend any intensified imaging surveillance scheme in the post-therapeutic setting. In analogy to what was shown for lung cancer screening, intensified surveillance may result in a survival benefit in high-risk groups, while it may be associated with a high rate of false-positive findings and an unnecessary exposure to ionizing radiation^30,31^.

For patients without exposure to extrinsic carcinogens, it could be discussed whether intensive post-treatment surveillance may be limited to 24 months, since most recurrences are diagnosed within this interval.

We show in a secondary analysis that an increase in SUV_max_ locally and regionally between two sequential posttherapeutic FDG-PETs is associated with a high risk of recurrence. An increase in SUV_max_ is therefore associated with a high suspicion of recurrent tumor.

If biopsies taken in this context are negative, false-negative results due to sampling error have to be ruled out. We observed only a few patients (10.6% local (36/337) and 7.7% regional (26/337)) with increasing SUV_max_ without proof of tumor recurrence. A constant or decreasing SUV_max_ on the other hand may be explained by inflammation and/or radionecrosis. The latter is sometimes very hard to distinguish from tumor recurrence on cross-sectional imaging alone. A previous study by our group showed that a combination of low SUV_max_ and location of the hottest voxel inside bone/cartilage enables differentiation from tumor recurrence^32^.

It should be noted that we intentionally used a rather hard cutoff (increase > 0 vs. stable/decrease ≤ 0) when performing our analysis. This provides clinicians a simple and easy rule for daily practice. However, although remaining unproven, one can assume that the risk of recurrence would be higher for a larger increase rather than a marginal increase of SUVmax. Interpretation in clinical context remains therefore necessary.

For the secondary analysis in which recurrence was detected following the second posttherapeutic FDG-PET at nine months, the majority of patients (22/40 (55.0%)) qualified for salvage surgery, meaning that the strategy of close follow-up with imaging is valuable. This rate compares favorably with salvage eligibility rates reported by other studies (23% to 38%)^23,33–35^. This finding, however, has to be interpreted with caution as eligibility for salvage surgery strongly depends on the primary tumor site, with the larynx being the site most often eligible for salvage surgery^26^. Previous studies did not report however on their imaging protocol during posttreatment surveillance. We assume that these did not include FDG-PET on regular intervals. Whether our higher salvage eligibility rate can be explained by FDG-PET monitoring remains unproven and should be investigated in future studies. This represents a limitation of our study. Although SUVmax uniquely identifies patients with recurrent disease, we did not show that FDG-PET provides information not obtainable by anatomic imaging (CT or MRI) alone.

Further, analysis of disease-specific survival showed no significant difference in patients with increasing vs. decreasing/stable SUVmax locally and regionally. This can be interpreted as lack of relevance of our findings, since increased SUVmax, although predicting local or regional recurrence, does not predict survival. However, maintaining high locoregional control rates are very important to avoid disfigurement, pain, and distress associated with locoregional failure.

Further limitations of our study need to be mentioned. First, it is a single center study and only one reader made the measurements, albeit in a standardized fashion, of FDG-PET metabolic parameters. This introduces a selection bias and reduces the external validity of the study. Second, we had a limited number of events and many censored events. This limits the strength of the statistical analysis, as we were not able to control for potential confounder (HPV status, extra nodal extension, etc.) in a multivariable analysis. Due to its retrospective design, the timing and interval of FDG-PET was slightly variable. We addressed this issue by allowing for a longer period of recurrence (between 8 and 14 months) for the secondary analysis. Further, we included all tumor sites of the head and neck except the nasopharynx and sinonasal area, which needs to be remembered when interpreting the salvages rates. Finally, we only measured SUVmax measurements of local tumor and dominant lymph node. Further parameters such as total lesion glycolysis (TLG) or metabolic tumor volume (MTV) were not assessed. However, in previous studies^8–10^ we showed that SUVmax was the metabolic parameters with the strongest predictive value.

## Conclusion

An increase in local and regional SUV_max_ between three and nine months after therapy is associated with an increased risk of tumor recurrence. SUV_max_ changes are a reliable tool for detecting tumor recurrence.

## Supplementary information

Supplementary file1

Supplementary file2

Supplementary file3

Supplementary file4
