# Supplementary figures and images for: Predictive value of suvmax changes between two sequential post-therapeutic FDG-pet in head and neck squamous cell carcinomas
Source: Sci Rep. 2020 Oct 7;10:16689. doi: 10.1038/s41598-020-73914-3 (PMC7542158; doi:10.1038/s41598-020-73914-3)

## Slide 1
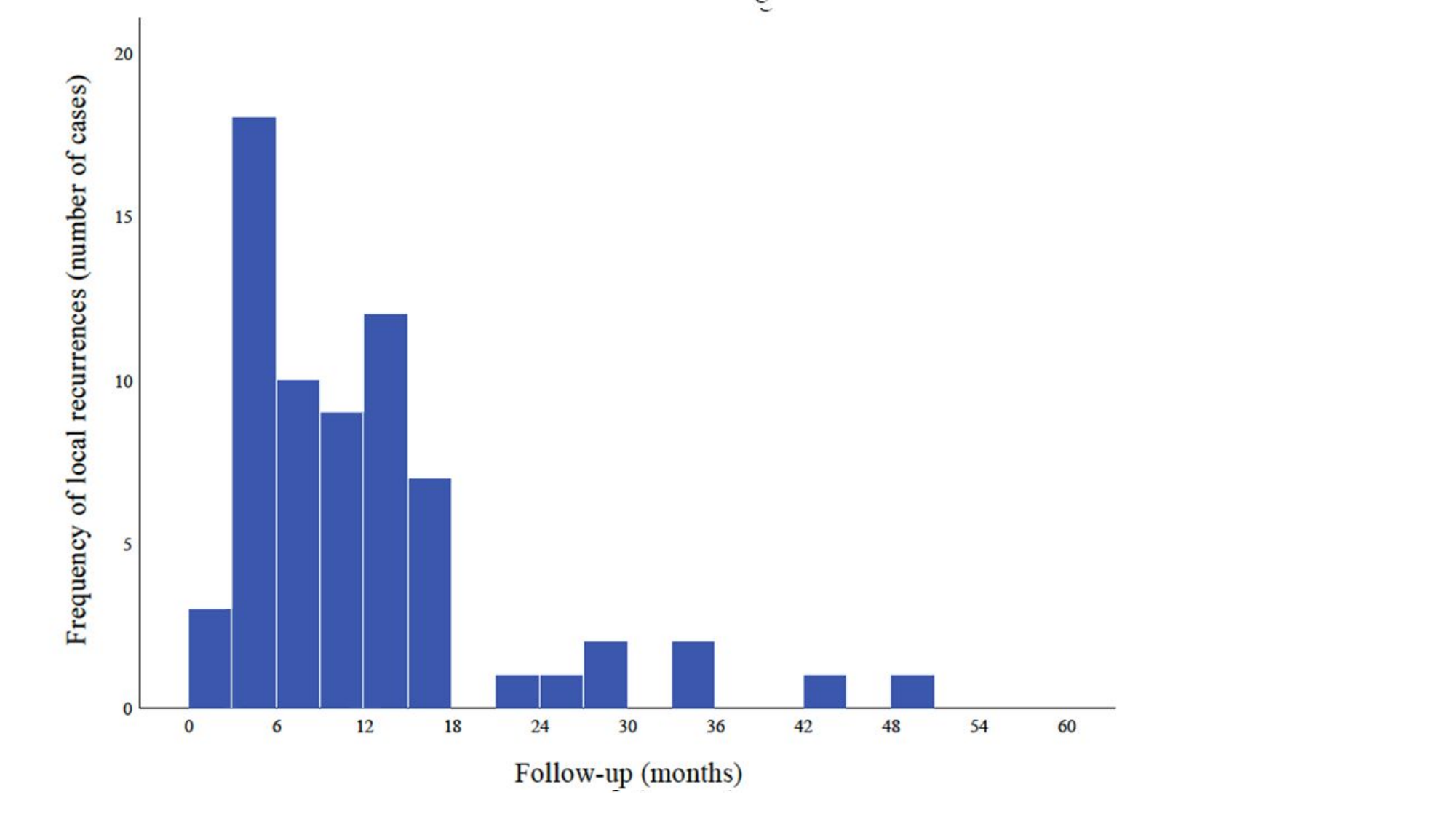

Supplement: Supplementary file 1 — Supplementary file1 [file 41598_2020_73914_MOESM1_ESM.pptx]

## Slide 1
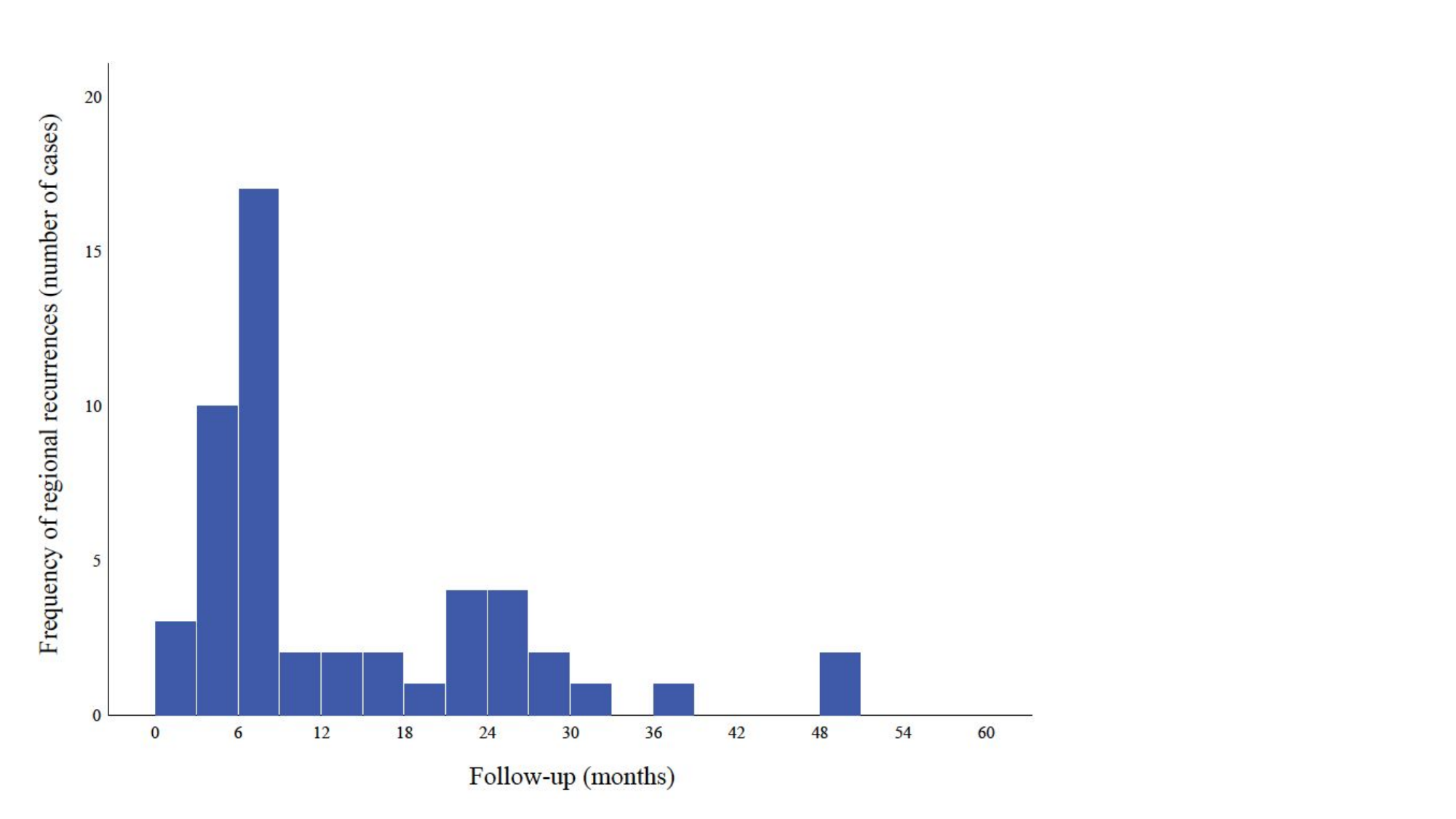

Supplement: Supplementary file 2 — Supplementary file2 [file 41598_2020_73914_MOESM2_ESM.pptx]

## Slide 1
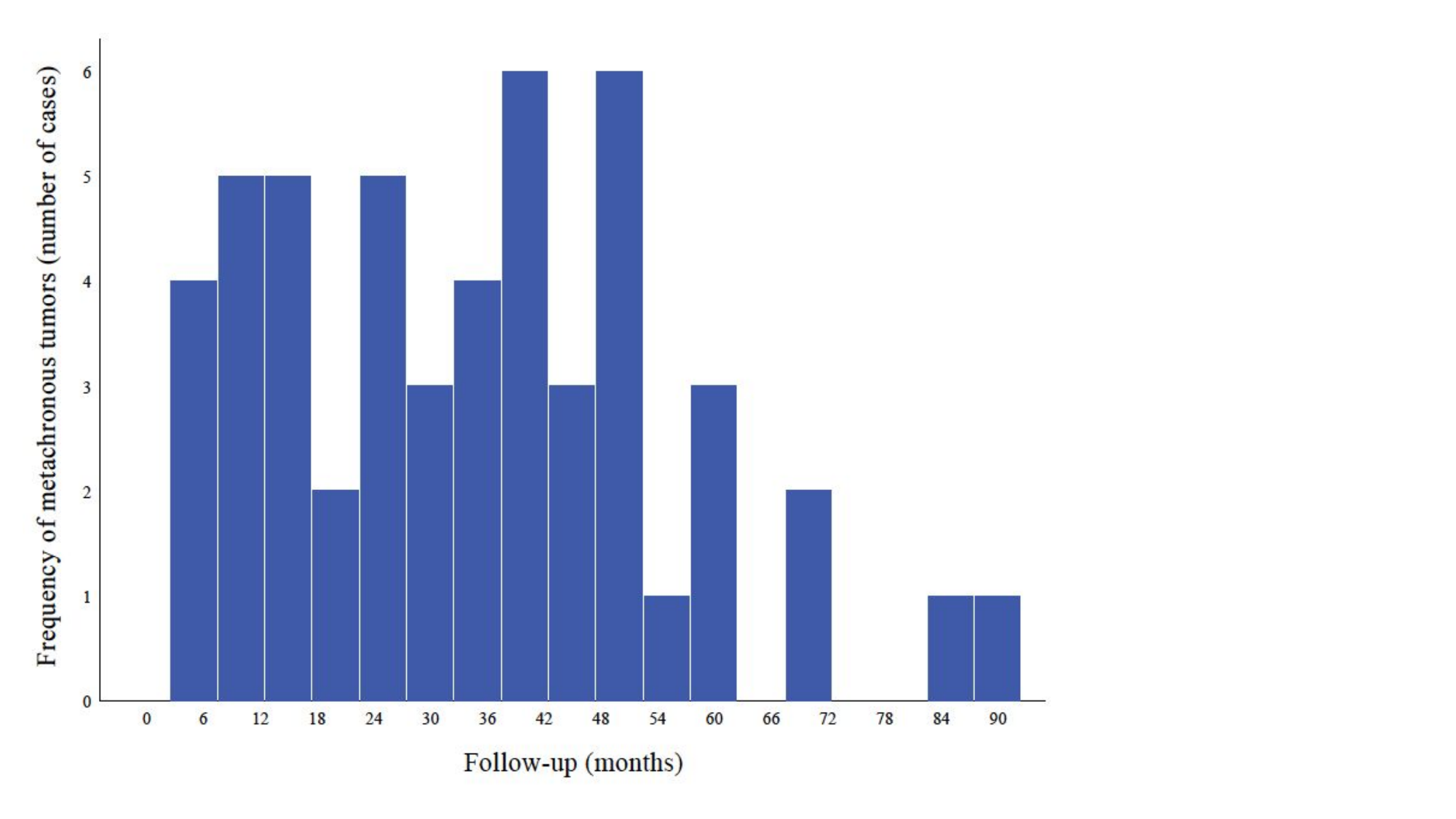

Supplement: Supplementary file 3 — Supplementary file3 [file 41598_2020_73914_MOESM3_ESM.pptx]
