# Supplementary material for: Predictive value of suvmax changes between two sequential post-therapeutic FDG-pet in head and neck squamous cell carcinomas
Source: Sci Rep. 2020 Oct 7;10:16689. doi: 10.1038/s41598-020-73914-3 (PMC7542158; doi:10.1038/s41598-020-73914-3)
Supplement: Supplementary file 4 — Supplementary file4 [file 41598_2020_73914_MOESM4_ESM.docx]

**PREDICTIVE VALUE OF SUVMax CHANGES between TWO Sequential Post-therapeutic FDG-PET IN head & Neck squamous cell carcinomas**

Thomas Stadler^1,2^, Martin W. Hüllner^2,3^, Martina A. Broglie^1,2^, Grégoire B. Morand^1,2^

^1^ Department of Otorhinolaryngology - Head and Neck Surgery, University Hospital Zurich, Zurich, Switzerland

^2^ University of Zurich, Zurich, Switzerland

^3^ Department of Nuclear Medicine, University Hospital Zurich, Zurich, Switzerland

***Corresponding Author**

Grégoire B. Morand

Department of Otorhinolaryngology - Head and Neck Surgery, University Hospital Zurich

Frauenklinikstrasse 24

CH-8091 Zurich

Switzerland

Tel: +41 44 255 58 50. Fax: +41 44 255 45 56.

Email: gregoire.morand@usz.ch

**Short title**

Predictive value of temporal changes in SUV_max_ in HNSCC

**Competing interests’ statement**

T.M.S, M.A.B. and G.B.M. have no competing interests or other interests that might be perceived to influence the results and/or discussion reported in this paper. M.W.H. has received research grants from GE Healthcare, a grant by Alfred and Annemarie von Sick for translational and clinical cardiac and oncological research and funds by the Artificial Intelligence in oncological Imaging Network of the University of Zurich.

**Keywords**

carcinoma, squamous cell; positron emission tomography; fluorodeoxyglucose F18; laryngectomy; organ preservation; tumor hypoxia; chemoradiotherapy
